# Adult Suicidality and Therapeutic Engagement: A Systematic Review

**DOI:** 10.1002/cpp.70170

**Published:** 2025-11-13

**Authors:** Chloé Muscat, Frédéric Verhaegen, Christophe Clesse

**Affiliations:** ^1^ INTERPSY Université de Lorraine Nancy France; ^2^ School of Psychology University of Roehampton London UK

**Keywords:** dropout, engagement, homework compliance, participation, suicide, treatment completion

## Abstract

**Background:**

Engagement in mental health care among adults with suicidality remains significantly low, with limited understanding of the factors influencing treatment retention and dropout.

**Aims:**

Exploring the factors influencing therapeutic engagement, such as attendance, active participation and completion of therapeutic tasks among adults with suicidality, could offer valuable insights for suicide prevention and management.

**Methods:**

This systematic review explored quantitative and qualitative studies examining barriers and/or facilitators to treatment engagement. Four main databases have been explored (PubMed, Embase, PsycInfo and Web of Science). Data were analysed and thematically organised with the help of a content analysis.

**Results:**

Eighteen studies focusing on adults experiencing various forms of suicidality, including suicidal ideation, behaviours, plans and attempts, have been included. Engagement is mainly perceived through appointment attendance, active participation or a multidimensional combination of factors. Logistical constraints (service availability, time and money) emerge as the most significant barrier to engagement, followed by stigma, treatment‐related experiences and beliefs and patient perception factors. No consensus between studies can be isolated regarding the impact of social support, severity of suicidal symptoms and psychiatric comorbidities. Trust in mental health professionals and interventions facilitating active patient participation positively influence treatment continuation.

**Conclusion:**

This review highlights the need to increase mental health funding to enhance service availability and improve the training of mental health professionals in suicide prevention and management. Developing integrative and empowering interventions fosters an essential climate of trust and hope in therapy that enhances engagement. Future research should prioritise study designs that distinguish between perceived and actual barriers to engagement to develop targeted interventions.

## Summary

- Actively addressing logistical barriers such as cost, time and service availability can support therapeutic engagement in adults experiencing suicidality.
- Exploring and acknowledging patients' previous treatment experiences, beliefs and experiences of stigma may help reduce barriers to engagement.
- Therapeutic engagement is enhanced when clinicians foster active patient participation and a sense of empowerment.

## 1 Introduction

Suicide, defined as the intentional act of ending one's own life, accounts for 1 in every 100 deaths worldwide. Notably, 58% of suicides occur before the age of 50, and suicide is the third leading cause of death among individuals aged 15–29 years (World Health Organization 2025). Beyond suicide itself, suicidality encompasses a spectrum of behaviours and thoughts associated with suicide risk: suicide attempts (non‐fatal acts with intent to die), suicidal ideation (thoughts of suicide, with or without suicidal intention), suicide plans (formulated decisions on how and when to act without active preparation) and preparatory suicidal behaviours (actions taken to prepare for suicide, such as acquiring means, without initiating the act itself) (De Leo et al. 2021). Young, elderly and, specifically, psychiatric patients (e.g., mood disorders, substance abuse and a history of suicide attempts) are more likely to experience suicidal behaviours (Kapur et al. 2006; Taylor et al. 2005; World Health Organization 2014). For instance, patients receiving psychiatric help have a suicide risk of 1.4% compared to 0.23% for patients in other medical services (Haute Autorité de Santé 2022). Exposure to suicide or suicidal behaviours can also lead to severe and lasting consequences for those experiencing suicidality as well as mental health professionals (Sandford et al. 2021), families and communities (Cerel et al. 2008). Among those particularly affected individuals, an increased risk of subsequent suicidal behaviour has been noted (Hill et al. 2020; Mehlum and Ramberg 2010). Due to the complex nature of suicidality, prevention strategies focusing on relational approaches, risk assessment and crisis management are the primary tools used by mental health practitioners (Ryan and Oquendo 2020). Among relational approaches, psychotherapy has been proven effective in preventing suicide and reducing suicidal symptoms (Sufrate‐Sorzano et al. 2023). However, help‐seeking perceived as one of the first steps in the direction of professional and relational support is often hindered by a low perceived need and stigma (Han et al. 2017).

One of the contemporary key aspects of suicidality prevention is to explore how therapeutic engagement, essential for successful therapy, could improve patients' outcomes. Former studies have indeed identified that therapeutic engagement could be a crucial factor for the continuation and discontinuation of mental health support for adults experiencing suicidality (Lebeau et al. 2013). Therapeutic engagement is defined as a therapeutic partnership between patient and staff, multifaceted, perceived as dynamic (fluctuating over appointments), co‐constructed (based on a trust relationship between the patient and the psychotherapist) (Bright et al. 2015) or dependent on patient characteristics (attendance at appointments, involvement and task completion) (Bijkerk et al. 2023; Tetley et al. 2011). Developing a set of observable indicators determining the quality of engagement could therefore be useful to improve psychotherapy effectiveness for suicide. Frameworks such as the Patient Health Engagement (PHE) model (Graffigna et al. 2017) and person‐centred care approaches (Ekman et al. 2012) have examined therapeutic engagement from different perspectives, particularly as an intrapsychic and motivational process. However, in suicidality research, these dimensions remain difficult to operationalise in empirical studies. Holdsworth et al.'s (2014) model is known to present a process model of therapeutic engagement where the client's motivation and therapeutic alliance determine the patient's engagement. Compared to other models, it offers a broad and dynamic conceptualisation of engagement, embedded in relational and motivational processes, while also providing observable and quantifiable indicators. Within this model, therapeutic engagement can be assessed through (i) attendance at appointments, (ii) active participation in discussions and (iii) completion of therapeutic tasks (Holdsworth et al. 2014).

**FIGURE 1:**
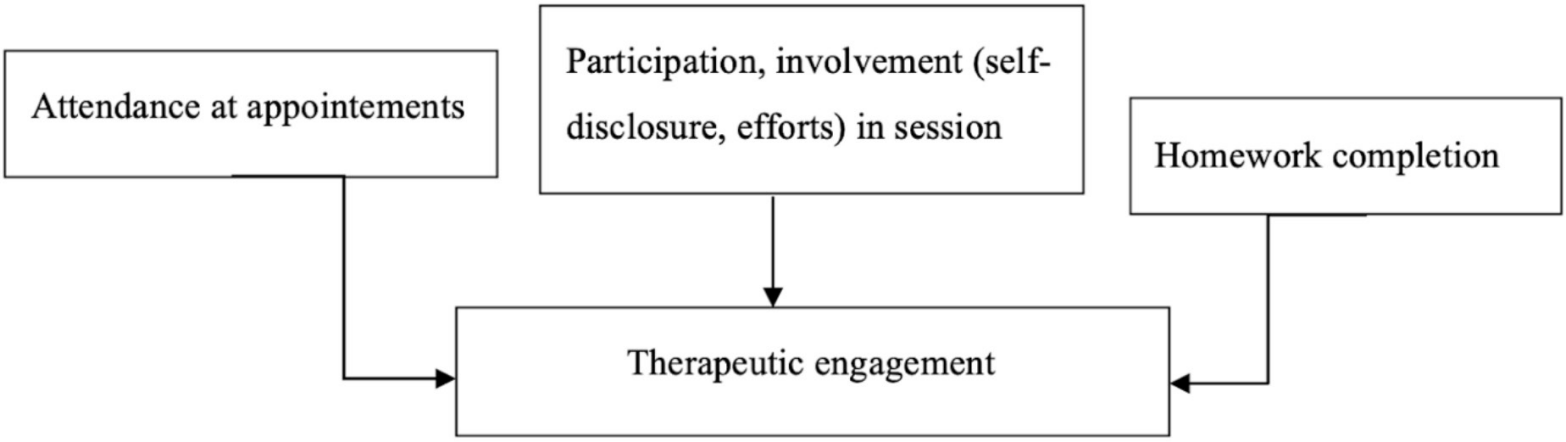
Applied model of therapeutic engagement (adapted from Holdsworth et al. 2014).

Based on Holdsworth et al.'s (2014) model, the main behavioural manifestations of engagement were isolated (Figure 1) to explore barriers and facilitators in clinical settings. This qualitative systematic review thus explores the barriers and facilitators affecting therapeutic engagement among adults experiencing suicidality. The objective of our study is to guide future development of targeted interventions for populations at risk of suicide while enabling clinicians to enhance patient engagement that provides improvements in therapeutic efficiency.

## 2 Method

### 2.1 Design

This qualitative systematic review aims to identify and discuss the influence of the barriers and facilitators that could affect therapeutic engagement among adults experiencing suicidality.

### 2.2 Search Strategy

Studies published in English were identified using relevant databases (PubMed, Web of Science, PsycInfo and Embase). The search period extended from 1 October 2023 to 31 March 2024 with no date restrictions applied. The English search terms and algorithm used were: suicid* AND (‘treatment engagement’ OR ‘reasons for drop* out’ OR ‘involvement’ OR ‘participation’ OR ‘attendance’ OR ‘treatment completion’ OR ‘homework compliance’ OR ‘in between tasks’ OR ‘self‐disclosure’ OR ‘activit* completion’) AND [full text] (‘barrier*’ OR ‘enhanc* factor*’ OR ‘influenc* factor*’ OR ‘facilitator*’) NOT (‘child*’ OR ‘ado*’ OR ‘youth’ OR ‘teen*’); applied on the abstract or title, except for the term mentioned in the full text. References of identified studies were also reviewed to ensure completeness, along with the ‘citation searching’ method.

### 2.3 Eligibility Criteria

Eligibility criteria were (i) publication written in English and French; (ii) participants aged 18 or older with suicidal ideation or behaviours (suicide attempts, death by suicide); (iii) participants aged 18 or older in direct contact with individuals having suicidal ideation or behaviours, such as mental health professionals or next‐of‐kin; (iv) studies where engagement is defined as attendance at appointments in mental health services (psychologist, psychiatrist, general practitioner for mental health reasons), participation, involvement and homework completion; (v) quantitative, qualitative and mixed‐methods designs including meta‐analyses, randomised controlled trials, cross‐sectional studies, cohort studies, case–control studies, structured or semi‐structured interviews and focus groups; (vi) examining factors influencing therapeutic engagement or disengagement (barriers, facilitators) in an initiated follow‐up.

Studies were excluded if they met the following criteria: (i) book chapters, opinion articles, editorials, clinical recommendations, case studies, research dissertations, theses and conference papers; (ii) participants younger than 18 years (children, adolescents); (iii) articles published in languages other than English or French; (iv) reference to the specificity of an intervention as a facilitator without mentioning components specifically influencing engagement; (v) references to intentions or behaviours solely related to help‐seeking.

### 2.4 Study Selection

**FIGURE 2:**
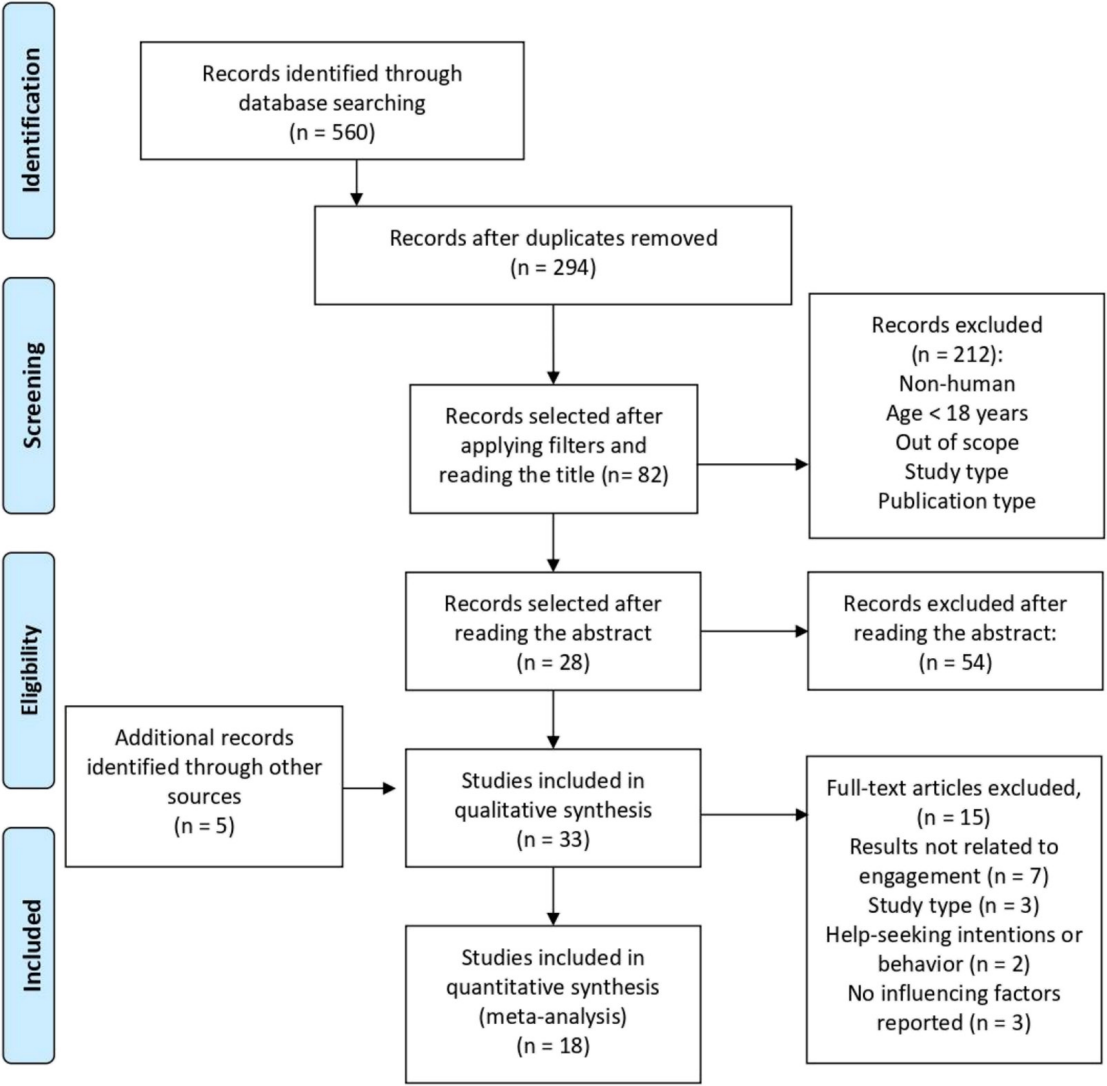
PRISMA flow chart (Liberati et al. 2009).

The selection and evaluation of studies were conducted in a double‐blind manner by C.M. and C.C. (Figure 2). A total of 560 studies were identified from the search strategy, followed by an exclusion of duplicates and a title screening evaluation that led to the abstract review of 82 studies. A total of 33 articles have been selected for a full‐text review (28 studies and 5 additional references). In total, 18 articles were included in the review.

### 2.5 Data Extraction

The following information was extracted from the included studies by the first author: author names; publication date; country of study; type and size of sample; age; percentage of female participants; type and design of the study; definition of therapeutic engagement; authors' conclusions; and any factors found to influence therapeutic engagement (socio‐demographic data, service utilisation data, behavioural variables and structural variables). All these data have been summarised in tables (Tables 1, 2, 3 and 4).

**TABLE 1:** Study characteristics.

No.	Authors (year) /Country	*N*	[Age range] Mean age (SD)	% women	Participants	Engagement component assessed	Study design	Data collection (data analysis)	Interpretations of the authors
1	Alonzo (2016) USA	36	39.07	80.6	Mental health professionals	Multidimensional (attendance, active participation, patient‐therapist connection)	Qualitative (qualitative description)	Focus group (thematic analysis)	Crucial role of the therapist in engaging clients.
2	Alonzo (2022) USA	22	33.45 (11.99)	50	Inpatient	Multidimensional attendance, participation	Qualitative (observational)	Semi structured decisional balance (thematic analysis)	At‐risk clients recognise the benefit of mental health services in meeting their problem‐solving, stress management and communication needs, but barriers such as doubt about the effectiveness of treatment may limit their engagement.
3	Alonzo and Colon (2020) USA	37	46.11	67.6	Inpatient	Attendance	Quantitative (retrospective observational)	Semi structured interviews (statistical analysis)	Presence of family support is associated with weak commitment, whereas a history of physical abuse is associated with stronger engagement.
4	Alonzo and Zapata Pratto (2021) Peru	32	N/A	93.5	Mental health professionals	Attendance	Qualitative (grounded theory)	Focus group (thematic analysis)	Lack of professional training can hinder engagement.
5	Büscher et al. (2022) International	1019	36	68	Suicidal individuals	Participation (modules completion)	Quantitative (meta‐analysis)	Data from quantitative studies (statistical analysis)	Importance of human support to improve adherence.
6	Costemale‐Lacoste et al. (2017) France	107	36.7 (14.5)	67	Outpatient	Attendance	Quantitative (naturalistic prospective)	Telephone survey (statistical analysis)	Making an appointment before discharge from emergency services facilitates short‐ and long‐term engagement.
7	Deuter et al. (2019) Australia	7	60–82	7.4	Elderly attempted suicide	Involvement (open communication)	Qualitative (phenomenological)	Semi‐structured interview (statistical analysis)	Social and professional support promotes engagement.
8	Glass et al. (2010) USA	71	N/A	N/A	Veterans attempted suicide.	Attending 3 sessions the first month and at least 2 sessions the next 2 months	Quantitative (naturalistic observation)	Medical records (descriptive and survival analyses)	Increasing the number of contacts promotes engagement.
9	Goldstone and Bantjes (2017) South Africa	18	N/A	N/A	Mental health professionals	Attendance	Qualitative (exploratory)	Focus group (thematic analysis)	Lack of professional training hinders patient engagement.
10	Hom et al. (2016) USA	483	36.39	10.4	Retired firefighters or in service with past suicidal experiences	Attendance	Quantitative (cross‐sectional)	Internet questionnaires (statistical analysis)	Firefighters with a history of suicidal ideation and behaviour were more likely to use mental health services than the general population. However, stigma and accessibility of care remained obstacles to treatment.
11	Ilardi and Kaslow (2009) USA	51	34.5	100	Women victim of domestic violence that attempted suicidal during the year	Attendance	Quantitative (observational)	Self‐administered questionnaires (statistical analysis)	Interpersonal difficulties promote attendance at appointments while fearful attachment reduces it.
12	Jerant et al. (2019) USA	44			People involved (health professionals, men who have committed suicide, relatives of people who have died by suicide, etc.)	Participation/disclosure	Qualitative (descriptive)	Focus group (thematic analysis)	Lack of professional training, masculine identity and fear of hospitalisation can impact the engagement of middle‐aged men.
13	Munasinghe et al. (2021) Australia	1654	18+	58.4	Patients at high risk of suicide referred to primary mental health care services	Attendance	Quantitative (observational retrospective)	Medical records and sociodemographic data (statistical analyses)	A younger age and a waiting time greater than 2 weeks are factors for disengagement.
14	Nichter et al. (2020) USA	84	49.4	17	War veterans with suicidal thoughts	Attendance	Quantitative (retrospective cross‐sectional)	Self‐administered questionnaires (statistical analyses)	Negative beliefs about mental health professionals and fear of harming one's reputation have been identified as major factors in non‐utilisation of care.
15	Riblet et al. (2019) USA	16	Low risk: 59.3 (8.3) High risk: 47.5 (8.6)	0	Hospitalised veterans referred for post‐hospitalisation follow‐up	Attendance (3 months after discharge)	Mixte (qualitative and quantitative, descriptive observation)	Semi‐structured interview and medical records analysis (thematic analysis, statistical analysis)	Predisposing, facilitating, medical and psychiatric characteristics influence engagement more than logistic variables.
16	Vitale et al. (2021) USA	126	55.73	31	Veterans	Attendance	Quantitative (cohort observational)	Self‐administered questionnaires (thematic analysis, statistical analysis)	Integrative interventions promote engagement.
17	Wray et al. (2019) USA	173	51.5	10	Post‐hospital suicidal veterans	Attendance	Quantitative (retrospective cross‐sectional)	Medical records analysis, telephone calls (statistical analysis)	Psychiatric history hinders engagement while telephone contacts and CBT‐type interventions promote it.
18	Zuromski et al. (2019) USA	168	N/A	N/A	Next‐of‐kin and supervisors' perspectives on soldier of US army die of suicide during years of services	Attendance	Quantitative (Psychological autopsy)	Structured interviews, comparisons of data from three groups (1 experimental group and two control groups) (statistical analysis)	Soldiers who died by suicide perceived more barriers to engagement than matched controls.

**TABLE 2:** Barriers and facilitators in qualitative and quantitative studies.

No.	Study	Barriers	Facilitators
**Qualitatives**			
1	Alonzo 2016	Client‐related variables: past treatment‐related experiences, fear of stigma, fear of consequences of expressing thoughts and emotions, feeling of hopelessness. Variables linked to the service: location and layout of the place, schedule and administration, lack of confidentiality, lack of collaboration between professionals. Variables linked to accessibility: housing instability, financial instability.	Variables linked to the intervention: normalise emotions and fears, psychoeducation, establishment of hope, giving the client the right to self‐determination. Therapist‐related variables: therapist authenticity. Variables linked to accessibility: financial stability Service‐related variables: collaboration between stakeholders. Client‐related variables: feeling safe, family support.
2	Alonzo 2022	Ineffectiveness: low perceived need for follow‐up combined with fear of ineffectiveness. Stigma: fear of being embarrassed to go to the therapist. Time: lack of time. Cost Psychological pain: difficulty confronting problems.	Acquire new skills: problem solving, coping strategies, communication skills. Source of support: place of listening and understanding, promotes emotional expression, secure place, place of affiliation with peers. Constructive feedback: hearing a new point of view gives new perspectives.
4	Alonzo and Zapata Pratto 2021	Meso‐level: lack of family structure, lack of awareness among parents, stigma. Macro‐level: illegality of suicide.	Meso‐level: family support Macro‐level: increase availability of services, community approaches.
7	Deuter et al. 2019		Active listening Accessibility Security and trust in the relationship Warm relationship and open communication
9	Goldstone and Bantjes 2017	Availability of services and lack of resources in services [interventions of lower quality than they should] Lack of professional skills Lack of financial resources Psychological trauma Low family support Stigma	
12	Jerant et al. 2019	Fear of incompetence of primary care clinicians: lack of training and sensitivity of clinicians regarding suicidal ideation; clinician's lack of understanding and sensitivity to suicidal ideation disclosure; lack of time and resources to discuss suicidal ideation in consultations. Fear of involuntary psychiatric hospitalisation Influence of masculine identity: social norms (normalisation of suicidal ideation, need for autonomy); stigma associated with expressing vulnerability	
15	Riblet et al. 2019	Increased severity of symptoms Lack of awareness of risk/low perceived need	
**Quantitatives**			
3	Alonzo and Colon 2020	Familial support	Physical abuses
5	Büscher et al. 2022	Employee	Social support Being a woman
6	Costemale‐Lacoste et al. 2017		Appointment booked before discharge
8	Glass et al. 2010	Greater number of hospitalisation	
10	Hom et al. 2016	Suicidal plan without attempt	Severity of suicidal behaviour: the more severe it is, the more commitment there is to care (suicidal ideation 68%, suicidal attempt 93%) Years of practice: The more years of practice, the more use of healthcare structures there is. Being older (35+) Higher annual income
11	Ilardi and Kaslow 2009	Fearful attachment style	Interpersonal worries (difficulties/stress in daily social interactions)
13	Munasinghe et al. 2021	Age 18–24 + 2 weeks before the first appointment	
14	Nichter et al. 2020	Older man	History of suicide attempt History of psychotrauma Diagnosis of depressive episode Multiple medical problems Be a woman Young age
15	Riblet et al. 2019		Patients with high perceived risk: more use and more intensely (5 or more appointments in the 3 months post‐hospitalisation).
16	Vitale et al. 2021		Intervention including multidimensional strategies focused on integrative and complementary medicine (reducing social isolation, promoting group cohesion, relaxation techniques (meditation, acupuncture), artistic activities, physical exercises, providing sleep and nutrition advice).
17	Wray et al. 2019	Substance abuse Greater number of hospitalisation	Post‐hospitalisation phone calls Participation in the post‐hospitalisation dialectical transition therapy group (psychoeducation, skills focused on crisis management)
18	Zuromski et al. 2019	Thinking the treatment is not working Thinking that problems can be handled alone Time, transportation or schedule constraints	

**TABLE 3:** Barriers grouped after thematic analysis.

Variables	Qualitative	Quantitative	Total
**Client‐related**			
Social support: *marital status, family, friends, relative, supervisor, chaplain, supportive or not, community approach, interpreter, need, perceived by others, opinion of relatives on treatment*.	4/9	3	3
Stigma: *fear of being hospitalised without consent, fear of being perceived as weak, fear of career being affected, fear of being perceived differently by peers or loved ones, fear of a negative impact, fear of stigma, fear of losing the trust of peers, feeling of shame, religious belief, feeling of illegality*.	1/2/4/9/12		5
Previous treatment experiences: *no experiences, bad experiences, terms of entry into care, non‐adherent, number of hospitalisations*.	1	8/17	3
Need, perceived and autonomy: *awareness of disorders, awareness of risk, feeling of getting better, feeling that stress is normal as a student, belief that problems can be managed alone, being described as having a lot of resources*.	15	18	2
Doubts about the effectiveness of the treatment: *non‐helpful, may cause more harm than good, no benefit*.	2	18	2
Trust in health professionals: *non‐invested therapist, lack of collaboration between professionals, weak professional support, lack of confidentiality*.	1/12		2
Hopelessness: *distress, hopelessness, difficulty talking about it, fear of being a burden*.	1/2		2
Attachment style: fearful attachment		11	1
Professional career: *being part of the Navy, the Air Force, being a volunteer firefighter*.		10	1
Age		13/14	2
Male gender: *social norms*	12		1
**Health‐related**			
Severity of suicidal symptoms: *suicidal ideation, suicide attempt, history of suicidal ideation or suicide attempts, worsening symptoms, plan with or without suicidal intention, duration of illness*.	15	10	2
Diagnoses/comorbidities: *substance abuse, alcohol dependence, depressive episode, post‐traumatic stress disorder, multiple medical conditions, anxiety, mood disorders, physical abuse*.	9	17	2
**Access‐related**			
Logistical constraints (time, money, work, means of transport): *time constraints, financial difficulties, transport difficulties, location of the place, being employed/unemployed*.	1/2/9/12	5/18	6
Availability of services: *lack of professionals, non‐qualitative treatment, waiting time to obtain an appointment*.	9/12	13	3

*Note:* The table is completed by referring to the corresponding study number.

**TABLE 4:** Facilitators grouped after thematic analysis.

Variables	Qualitative	Quantitative	Total
**Client‐related**			
Social support: *marital status, family, friends, relatives, supervisors, supportive chaplains, community approach, interpreters, need perceived by others, referral by a loved one*.	1/4	5/11	4
Need, perception and autonomy: *awareness of disorders and risk of relapse*.		15	1
Professional career: *being part of the navy, army, air force, number of years of service, volunteer firefighter*.		10	1
Trust in health professionals: *confidence in establishing a relationship and connection, invested therapist, collaboration between professionals, professional support, authenticity, positive attitudes towards mental health professionals*	1/2/7		3
Age		10/14	2
Female gender		5/14	2
**Intervention‐related**			
Booking an appointment before discharge		6	1
Phone calls		17	1
Acquisition of skills: *new coping strategies, new communication skills, new point of view, new perspectives, being flexible and creative, psychoeducation, normalising emotions …*	1/2	16/17	4
**Access‐related**			
Accessibility and logistical aspects (time, money, means of transport): *financial stability, location and layout of the place*.	1/7	10	3
Availability of services	4		1
**Health‐related**			
Severity of suicidal symptoms: *suicidal ideation, suicide attempt, history of suicidal ideation or suicide attempts, worsening symptoms, plan with or without suicidal intention, duration of illness*.		10/14	2
Diagnoses/comorbidities: *substance abuse, alcohol dependence, depressive episode, post‐traumatic stress disorder, multiple medical conditions, anxiety, mood disorders, physical abuse*.		3/14	2

*Note:* The table is completed by referring to the corresponding study number.

### 2.6 Data Analysis

Data analysis was performed according to the Preferred Reporting Items for Systematic Reviews and Meta‐Analyses (PRISMA; Page et al. 2021) and the Synthesis Without Meta‐analysis (SWiM) guidelines (Campbell et al. 2020). Following SWiM recommendations, data from quantitative, qualitative and mixed‐methods studies were first extracted separately. Quantitative data were summarised using vote counting based on the direction of effect, qualitative data were organised and analysed using inductive content analysis to identify recurring themes (Elo and Kyngäs 2008) and a convergent synthesis design was then applied to integrate findings across study types for narrative synthesis.

### 2.7 Bias Evaluation

The Joanna Briggs Institute (JBI) (Moola et al. 2020) was used in a double‐blind manner by the first author (C.M.) and third author (C.C.) to assess the biases of the included studies (Appendix A).

## 3 Results

Eighteen studies (9 quantitative, 8 qualitative and 1 mixed method) with cross‐sectional, observational, cohort, quasi‐experimental designs and one psychological autopsy were included (Table 1). Most of the studies (*n* = 16) were conducted in high‐income, Westernised countries (12 in the United States, 1 international, 2 in Australia and 1 in France) with predominantly White populations. Only two studies (South Africa and Peru) offer a diverse perspective. Sample sizes range from 7 to 1654 participants, aged between 18 and 82 years. Gender distribution varied across studies with a prevalence of women ranging from 10.4% to 100%. The included studies examined the perspectives of different groups affected by suicidality: 3 studies focused on mental health professionals' views; 5 studies involved hospitalised (*N* = 3) or outpatient patients (*N* = 2); 3 studies included individuals experiencing suicidality; 6 studies examined military personnel or veterans; 1 study focused on women victims of violence; and 1 study explored the perspectives of firefighters. In spite of a lack of information on the theoretical perspective surrounding the notion of therapeutic engagement adopted by the researchers and its consistency with the methodological choices, most studies demonstrate acceptable to good levels of bias.

### 3.1 Evaluation of Therapeutic Engagement's Variables

Therapeutic engagement evaluation mainly relies on diverse methodologies. In most studies (*n* = 13), therapeutic engagement components are only assessed by attendance at appointments. Only two studies (Alonzo 2016, 2022) evaluate engagement multidimensionally, considering the client–therapist relationship (warmth, open communication, emotional connection), client‐related variables (attendance, involvement, trust in the therapist) and a therapist‐specific variable (understanding the patient's hopes for the treatment). Three studies assess participation and involvement in interviews based on the number of modules completed (Büscher et al. 2022), disclosure of suicidal ideation (Jerant et al. 2019) and the degree of openness in the therapeutic relationship (Deuter et al. 2019).

### 3.2 Client‐Related Variables

Clinical variables play a significant role in therapeutic engagement. Participants with a history of suicide attempts appear to be more engaged in healthcare (Hom et al. 2016; *p* < 0.001; Nichter et al. 2020) when compared to those presenting only suicidal plans (Hom et al. 2016). Particular attention to the evolution of suicidal expression and symptoms should be observed as a worsening symptomatic expression is seen as a crucial disengagement factor (Riblet et al. 2019). In addition, one cross‐sectional study found that multiple medical problems and a diagnosis of a depressive episode facilitate continued care (*p* = 0.04 and *p* < 0.001; Nichter et al. 2020). From a retrospective point of view, psychological trauma (Goldstone and Bantjes 2017) and substance abuse (b = 0.83, SE = 0.30, *p* = 0.006; Wray et al. 2019) act as barriers to engagement. Contrasted results, however, consider that past psychological trauma (OR = 1.13, 95% CI [1.02–1.26], *p* < 0.01; Nichter et al. 2020), including physical abuse (*r* = −0.384; *p* = 0.019; Alonzo and Colon 2020), could also be a therapeutic engagement facilitator. Potential biases—such as reliance on self‐reported data, small sample sizes and limited generalisability—suggests that these findings should be interpreted with caution. Further research is needed to confirm the impact of past adverse health outcomes, considering their complexity and various interactions with mediating factors.

Past negative experiences and emotional‐relational characteristics also affect therapeutic engagement. Indeed, previous negative treatment experiences (Alonzo 2016), a higher number of hospitalisations (CI = 3.56–20.73, *p* < 0.01; Glass et al. 2010; b = 0.22, SE = 0.10, *p* < 0.02; Wray et al. 2019) and doubts about treatment efficacy (Alonzo 2022; Zuromski et al. 2019) have been associated with therapeutic disengagement among patients facing suicidality. Similarly, feelings of hopelessness (Alonzo 2016), difficulties in facing and discussing problems (Alonzo 2022) and a fearful attachment style characterised by discomfort with intimacy and dependency on others (*F* = 53.497, *p* < 0.05; Ilardi and Kaslow 2009) are all associated with higher risks of therapeutic dropout.

Stigma and self‐awareness have also been identified as relevant factors for therapeutic engagement. Stigma was identified as a barrier to engagement in five qualitative studies, primarily from the perspective of mental health professionals (*N* = 4), encompassing fears of disclosing suicidal ideation (Alonzo 2016), embarrassment about therapy (Alonzo 2022), family stigma linked to the illegality of suicide (Alonzo and Zapata Pratto 2021), fear of appearing vulnerable (Jerant et al. 2019) and general stigma (Goldstone and Bantjes 2017). In parallel, a patient's lack of awareness related to its own suicidal behaviours (Riblet et al. 2019) as well as the patient's desire to handle problems on its own (Zuromski et al. 2019) is considered a barrier to therapeutic engagement. Conversely, patients' greater risk awareness is associated with more intensive use of mental health services, indicated by a higher number of attended appointments (Riblet et al. 2019).

Finally, demographic variables offer contrasted perspectives regarding their potential influence on therapeutic engagement. Being a female seems associated with better therapeutic engagement. Indeed, female gender appeared as a facilitator in two quantitative studies (*p* = 0.049; Büscher et al. 2022; Nichter et al. 2020) while male gender is identified as a barrier in two other studies (Jerant et al. 2019; *F* = 4.77, *p* = 0.02; Nichter et al. 2020). The design of these studies did not address other gender expressions. However offers a more contrasted perspective as being less than 24 years old has been identified by an observational study including a large sample (*N* = 1654) as a barrier to therapeutic engagement (OR = 2.18, 95% CI = [1.47, 3.23]), (Munasinghe et al. 2021). However, another multivariate comparative study found older age to be a barrier and younger age a facilitator (*F* = 9.31, *p* = 0.002), (Nichter et al. 2020).

### 3.3 Social Support, Accessibility and Interventions‐Related Variables

Social support is another category of factor widely explored and associated with therapeutic engagement for adults facing a suicidality crisis. First, quantitative analyses found a significant association between human support and engagement (b = 0.228; *n* = 486; k = 5; Büscher et al. 2022) as well as between difficulties in social interactions and higher treatment attendance (*F* = 2.878, *p* < 0.05; Ilardi and Kaslow 2009). Second, unbalanced family support, whether it is a lack of family support (Alonzo and Zapata Pratto 2021; Goldstone and Bantjes 2017) or very important family support (*r* = −0.324; *p* = 0.05; Alonzo and Colon 2020) has hindered engagement. An exploration of potential reverse causality in the study, specifically the suggestion that strong family support is linked to hindered engagement, has yet to be conducted to clarify this factor. This could clarify findings from qualitative studies identifying that balanced family support (Alonzo 2016; Alonzo and Zapata Pratto 2021) and social support from group therapy (Deuter et al. 2019) are perceived as facilitators of therapeutic engagement.

Similarly, logistical constraints negatively impact therapeutic engagement. Identified factors are (i) a limited infrastructure availability leading to long waiting times before the first appointment (OR = 1.69, 95% CI = [1.18, 2.43]) (Munasinghe et al. 2021); (ii) a lower quality of interventions (Goldstone and Bantjes 2017; Jerant et al. 2019); (iii) time constraints (Alonzo 2016, 2022; Zuromski et al. 2019); (iv) being unemployed (*p* = 0.007) (Büscher et al. 2022); (v) the high perceived cost of therapy (Alonzo 2016, 2022; Goldstone and Bantjes 2017); (vi) transportation difficulties to the consultation site (Alonzo 2016; Zuromski et al. 2019). Conversely, financial stability (Alonzo 2016), higher annual income (Hom et al. 2016) and greater service availability (Alonzo and Zapata Pratto 2021; Deuter et al. 2019) are all considered therapeutic engagement facilitators while preserving care retention.

Finally, trust as well as caring and self‐empowering approaches provided by healthcare professionals are crucial for engagement in therapy. Qualitative studies have identified that concerns about confidentiality (Alonzo 2016), a lack of therapist training as well as a lack of sensitivity (Jerant et al. 2019) are perceived as disengagement facilitators. On the contrary, attentive, authentic and warm professionals (Alonzo 2016, 2022; Deuter et al. 2019) encourage patients to continue therapy while maintaining engagement. In addition, developing new skills through psychoeducation (Alonzo 2016; Wray et al. 2019), problem‐solving, communication methods (Alonzo 2022) or integrative interventions (*p* = 0.0052); (Vitale et al. 2021) helped promote engagement. Phone calls (Wray et al. 2019) and scheduling appointments before discharge (*p* = 0.008; Costemale‐Lacoste et al. 2017) also had a positive effect.

## 4 Discussion

This systematic review identified four categories of variables known to impact therapeutic engagement related to suicidality. On one hand, stigma and negative belief as well as logistical constraints factors emerge as the biggest barriers to therapeutic engagement. On the other hand, social support and trust in health professionals as well as intervention‐related factors targeting skills acquisition are the strongest facilitators. The findings also highlight inconsistent measurement of engagement across studies due to the fact that most research focuses on a single dimension of therapeutic engagement, failing to capture its complexity, as highlighted by models like Holdsworth et al. (2014). Lastly, included studies predominantly reflect high‐income, Westernised countries, underscoring the need for more culturally diverse research to allow generalisability.

Our analysis demonstrates that stigma is frequently identified as a primary barrier to therapeutic engagement, despite contrasting perspectives presented in other studies. For instance, the 2011 World Health Organisation survey found that stigma negatively affected only 7% of individuals experiencing suicidal thoughts (Hom et al. 2015). Similarly, Ammerman et al. (2021) reported comparable levels of stigma among individuals both engaged in and not engaged in care. These findings suggest that stigma may operate more as a perceived barrier than as a direct cause of disengagement. Individuals with mental health conditions may internalise and amplify the stigma associated with their diagnoses, leading to self‐stigmatisation and an anticipatory fear of rejection—even when such fears are not necessarily reflective of the actual attitudes of society or healthcare professionals. Moreover, recent evidence suggests that individual stigma (self‐stigma) is a stronger predictor of suicide risk than public or societal stigma (Oexle et al. 2018). This raises the possibility that a substantial proportion of disengagement attributed to stigma may, in fact, be more closely related to self‐stigmatisation and negative self‐perceptions than to overt discriminatory behaviour. A more nuanced examination of different forms of stigma—such as self‐stigma, familial stigma and stigma from healthcare providers—would be valuable for understanding their impact on therapeutic engagement. Quantifying disengagement rates associated with each type of stigma may enable clinicians to develop more targeted, person‐centred interventions that actively promote engagement, such as safety planning and collaborative strategies to support treatment adherence and reduce suicide‐related distress (Ferguson et al. 2022).

Second, negative self‐beliefs and negative beliefs about treatment are also identified as key barriers to therapeutic engagement. These aspects can be targeted early in any form of intervention, whether professional or supportive, in order to foster hope and dismantle self‐imposed barriers (Schechter et al. 2013). It is equally important to maximise facilitators of engagement as early as possible, thereby creating leverage over other potential obstacles. The development of new skills, supported by integrative interventions that combine psychoeducation with the completion of psychotherapeutic tasks (Büscher et al. 2022; Wray et al. 2019), may counteract these negative beliefs. Repositioning the patient as an active participant in their care can thus be highly effective in promoting continued engagement. Cognitive and behavioural therapies are grounded in this principle, often incorporating therapeutic tasks both during and between sessions. Clinicians should therefore prioritise activities that encourage behavioural engagement. For instance, patients might be invited to complete their suicide risk prevention plan during a session, while also being asked about the knowledge or strategies they already possess. This active involvement, in collaboration with the therapist, promotes a sense of empowerment and thereby facilitates therapeutic engagement.

Logistical constraints, as identified in our results, also represent a significant barrier to therapeutic engagement that needs to be addressed. This broad factor is intrinsically linked to the lack of financial resources allocated to mental health services, which hampers both the availability and quality of care (Goldstone and Bantjes 2017; Jerant et al. 2019; Munasinghe et al. 2021). According to a 2013 WHO report, annual funding for mental health worldwide accounted for only 0.5% to 5% of total health expenditures (World Health Organisation 2013). This lack of public funding continues to force 17% of countries to place the cost of mental healthcare almost entirely on individuals, thereby compromising their access to care (World Health Organisation 2017). In France, despite the existence of Crisis Intervention Units (known as ‘CAC’ in French) aimed at addressing psychiatric emergencies, their territorial coverage remains insufficient, particularly in low‐income areas where suicide risk factors are heightened. Reforms targeting health policies are therefore necessary to improve the territorial coverage of these units, ensuring effective secondary prevention and facilitating rapid access to nearby care. Primary prevention should also be strengthened by holding primary care professionals, such as general practitioners, more accountable. They should systematically assess suicide risk and promote national 24/7 hotlines. Combined with awareness campaigns, these actions would encourage the early detection of suicidal thoughts and effective referral to specialised services. Concurrently, health funding should be directed towards more comprehensive training for professionals, particularly in intercultural competencies, to optimise therapeutic alliances with all populations, regardless of their origins and backgrounds (Ilozumba et al. 2022). By reinforcing equitable funding for suicide prevention and mental healthcare across territories, public authorities could overcome significant logistical and financial barriers to therapeutic engagement.

Our review also establishes that social support, depending on its modalities, is a facilitator as well as a barrier for therapeutic engagement. In the literature social support is more frequently identified as a facilitator of engagement in mental health services than as a barrier (Kleiman and Liu 2013; Hansen et al. 2018; Kim et al. 2012). This complexity can be attributed to the multifaceted nature of social support. Emotional support and compassionate listening can encourage engagement, whereas an uninformed social circle may discourage or even oppose it due to misunderstanding. Additionally, the shame and stigma associated with suicidal thoughts can lead some individuals to conceal their condition from their surroundings, thereby depriving themselves of potential support. Clinically, strong social or family support can have both positive and negative effects on engagement, depending on the level of involvement from relatives. In contrast, social isolation can impede engagement due to the absence of a support system in the patient's daily life. Cross‐referencing data on individuals' social situations, self‐stigmatisation, and their engagement in care could refine the understanding of these complex interconnections. Offering psychoeducation programmes, such as BREF, to relatives could help them better understand their loved ones' suicidality, thus providing greater support. This, in turn, would enable patients to develop a sense of belonging and potentially reduce suicidal ideation.

Trust in healthcare professionals, such as feeling safe and supported, experiencing collaboration between professionals, and perceiving therapist authenticity, is a key facilitator of therapeutic engagement. Accordingly, the physical environment of the consultation room must be carefully managed, as patients' first impressions of this space play an important role in establishing a connection with the caregiver (Jones 2020). Creating an empathetic, compassionate and non‐judgemental atmosphere is essential to encourage the disclosure of suicidal thoughts and reduce the self‐stigmatisation often associated with them (Hom et al. 2017). This, in turn, enables the therapist to conduct an accurate risk assessment within a secure and containing environment. Providing patients who express suicidal thoughts with a card listing emergency contact numbers is a recommended practice. Beyond its practical function, this gesture symbolises the professional's attentive listening, empathy, and willingness to offer support. It reflects care and a commitment to ensuring continuity of care during potential crises. Such an approach not only strengthens the therapeutic alliance and trust between patient and clinician but also equips the individual with vital resources for use between sessions.

Given the current state of scientific knowledge, it remains premature to determine whether the severity of suicidal symptoms and other psychiatric comorbidities constitute barriers to therapeutic engagement. To better account for this complexity, cross‐sectional studies that incorporate multiple interacting factors are required. Such research would enable a more nuanced understanding of engagement and, ultimately, inform the development of more appropriately tailored clinical interventions.

Lastly, the lack of clear and consistent definitions of engagement in the literature must be addressed to improve its assessment. Models such as Holdsworth et al. (2014), used in this review, should be further developed as they offer practical, measurable frameworks that reflect the concept's complexity. Future research should prioritise data from individuals with suicidal thoughts or behaviours, rather than focusing mainly on professionals' perspectives. This would offer new clinical insights and improve the understanding of patient‐specific barriers. Key recommendations to research and practice would therefore be supported by the establishment of a shared definition of therapeutic engagement, taking into account its complexity. Second is a stronger focus on cultural diversity, equally ensuring that support structures are implemented in low‐income settings and providing comprehensive training in suicide risk prevention and intercultural competence to professionals involved in mental health care. Third, active patient participation should be encouraged, with efforts aimed at addressing both client‐related and intervention‐related barriers to engagement. Our findings underscore the importance of involving patients as active agents in their treatment, as participatory approaches and skill development have been shown to enhance engagement (Alonzo 2016, 2022; Wray et al. 2019; Vitale et al. 2021). Similarly, Tuvesson et al. (2025) highlight that recovery is facilitated when individuals have opportunities to pursue empowerment and growth within an authentic and engaging therapeutic environment. This convergence of evidence suggests that therapeutic engagement is strengthened when patients are both supported by attentive, authentic professionals and encouraged to cultivate autonomy and active participation in their care.

### 4.1 Strengths and Limitations

This qualitative systematic review capitalised on several advantages that contribute to the reliability of our results. The study protocol was grounded in a robust conceptualisation and operationalisation of the definition of therapeutic engagement, which enabled the inclusion of a diverse range of studies and facilitated a comprehensive exploration of the concept. Additionally, the representativeness of the samples supports the generalisation of the findings. Notably, this is the first systematic review on this topic, which raises new questions and directions for future research regarding therapeutic support. However, several limitations must be acknowledged. First, the inclusion of studies based on multiple and occasionally incomplete definitions of engagement may introduce a potential source of bias, which could affect the validity of the results. Consequently, we recommend that future studies replicate this work and establish a clear, consistent definition of therapeutic engagement before attempting to generalise the findings. Furthermore, although Holdsworth et al.'s (2014) model provides a useful and integrative framework for operationalising therapeutic engagement, its simplified structure may not fully capture the complexity of active engagement in clinical practice, highlighting the need for complementary models in future research. The limited consideration of inclusion factors such as disability, gender, sexual orientation and cultural background also restricts the broader generalisation of these findings. Future studies should quantitatively and qualitatively assess the influence of each of these dimensions, for example through frameworks such as the Social GGRRAAACCEEESSS model (Burnham 2012), while adopting an intersectional perspective to account for interactions between identity markers.

## 5 Conclusions

Interventions that enable patients to take an active role, such as the development of knowledge and skills and active involvement in their care, promote engagement. Conversely, factors such as stigma, previous experiences, and other patient‐related variables can negatively influence engagement. Clear operational definitions of engagement are essential to effectively observe and measure it, thus allowing healthcare professionals to design appropriate interventions for patients at risk of suicide. Furthermore, future research must aim to better represent cultural diversity to ensure that conclusions are applicable to all populations.

## Conflicts of Interest

The authors declare no conflicts of interest.

## Data Availability

Data sharing is not applicable to this article as no datasets were generated or analysed during the current study.

## Appendix A Table for Assessing the Quality of Studies Using the JBI Checklists

**Table:**

Cross‐sectional analytical studies								
	1. Were the criteria for inclusion in the sample clearly defined?	2. Were the study subjects and the setting described in detail?	3. Was the exposure measured in a valid and reliable way?	4. Were objective, standard criteria used for measurement of the condition?	5. Were confounding factors identified?	6. Were strategies to deal with confounding factors stated?	7. Were the outcomes measured in a valid and reliable way?	8. Was appropriate statistical analysis used?
3. Alonzo and Colon (2020)	Unclear	Yes	Unclear	Yes	Yes	Unclear	Yes	Yes
8. Glass et al. (2010)	Yes	Yes	Yes	Yes	Yes	Yes	Yes	Yes
10. Hom et al. (2016)	Yes	Yes	Yes	Yes	Yes	Unclear	Yes	Yes
11. Ilardi and Kaslow (2009)	No	Yes	No	No	Yes	Yes	No	Yes
13. Munasinghe et al. (2021)	Yes	Yes	Yes	Yes	Yes	Yes	Yes	Yes
14. Nichter et al. (2020)	Yes	Yes	Unclear	Yes	Yes	Yes	Yes	Yes
15. Riblet et al. (2019)	Yes	Yes	Cannot tell	Cannot tell	Yes	No	Yes	Yes
17. Wray et al. (2019)	Yes	Yes	Yes	Yes	Unclear	Unclear	Yes	Yes

Cohort studies											
	1. Were the two groups similar and recruited from the same population?	2. Were the exposures measured similarly to assign people to both exposed and unexposed groups?	3. Was the exposure measured in a valid and reliable way?	4. Were confounding factors identified?	5. Were strategies to deal with confounding factors stated?	6. Were the groups/participants free of the outcome at the start of the study (or at the moment of exposure)?	7. Were the outcomes measured in a valid and reliable way?	8. Was the follow up time reported and sufficient to be long enough for outcomes to occur?	9. Was follow up complete, and if not, were the reasons to loss to follow up described and explored?	10. Were strategies to address incomplete follow up utilised?	11. Was appropriate statistical analysis used?
6. Costemale‐Lacoste et al. (2017)	Yes	Yes	Yes	Yes	Yes	Yes	Yes	Yes	Unclear	Cannot tell	Yes
16. Vitale et al. (2021)	Unclear	Cannot tell	Cannot tell	Unclear	Unclear	Cannot tell	Yes	Yes	Yes	Cannot tell	Yes

Case–control studies								
	1. Were patient's demographic characteristics clearly described?	2. Was the patient's history clearly described and presented as a timeline?	3. Was the current clinical condition of the patient on presentation clearly describe?	4. Were diagnostic tests or assessment methods and the results clearly described?	5. Was the intervention(s) or treatment procedure(s) clearly described?	6. Was the post‐intervention clinical condition clearly described?	7. Were adverse events (harms) or unanticipated events identified and described?	8. Does the case report provide takeaway lessons?
18. Zuromski et al. (2019)	Yes	Unclear	Unclear	Yes	Unclear	Unclear	Unclear	Yes

Systematic review											
	1. Is the review question clearly and explicitly stated?	2. Were the inclusion criteria appropriate for the review question?	3. Was the search strategy appropriate?	4. Were the sources and resources used to search for studies adequate?	5. Were the criteria for appraising studies appropriate?	6. Was critical appraisal conducted by two or more reviewers independently?	7. Were there methods to minimise errors in data extraction?	8. Were the methods used to combine studies appropriate?	9. Was the likelihood of publication bias assessed?	10. Were recommendations for policy and/or practice supported by the reported data?	11. Were the specific directives for new research appropriate?
5. Büscher et al. (2022)	Yes	Yes	Yes	Yes	Yes	Yes	Yes	Yes	Unclear	Yes	Yes

Qualitative studies										
	1. Is there congruity between the stated philosophical perspective and the research methodology?	2. Is there congruity between the research methodology and the research question or objectives?	3. Is there congruity between the research methodology and the methods used to collect data?	4. Is there congruity between the research methodology and the representation and analysis of data?	5. Is there congruity between the research methodology and the interpretation of results?	6. Is there a statement locating the researcher culturally or theoretically?	7. Is the influence of the researcher on the research, and vice‐ versa, addressed?	8. Are participants, and their voices, adequately represented?	9. Is the research ethical according to current criteria or, for recent studies, and is there evidence of ethical approval by an appropriate body?	10. Do the conclusions drawn in the research report flow from the analysis, or interpretation, of the data
1. Alonzo (2016)	Yes	Yes	Yes	Yes	Yes	Cannot tell	Yes	Yes	Yes	Yes
2. Alonzo (2022)	Yes	Yes	Yes	Yes	Yes	No	Yes	Yes	Yes	Yes
4. Alonzo and Zapata Pratto (2021)	Unclear	Yes	Yes	Yes	Yes	No	Yes	Yes	No	Yes
7. Deuter et al. (2019)	Unclear	Yes	Yes	Yes	Yes	Cannot tell	Cannot tell	Yes	Yes	Yes
9. Goldstone and Bantjes (2017)	Yes	Yes	Yes	Yes	Yes	Yes	Yes	Yes	Cannot tell	Yes
12. Jerant et al. (2019)	Unclear	Yes	Yes	Yes	Yes	Unclear	Yes	Yes	Yes	Yes
15. Riblet et al. (2019)	Cannot tell	Yes	Yes	Yes	Yes	Unclear	Unclear	Yes	Yes	Yes
